# Awareness of Healthy Lifestyle Among Elderly Population During Aging in Al-Ahsa, Saudi Arabia

**DOI:** 10.7759/cureus.49054

**Published:** 2023-11-19

**Authors:** Danah S Alali, Abdulelah A Alshebly, Ajlan Alajlani, Arwa H Al Jumaiei, Zahra M Alghadeer, Sayed Ibrahim Ali

**Affiliations:** 1 College of Medicine, King Faisal University, Al-Ahsa, SAU; 2 Family and Community Medicine, King Faisal University, Hofuf, SAU

**Keywords:** alahsa, saudi arabia, geriatrics, healthy lifestyle, elderly awareness

## Abstract

Introduction: The research explores healthy aging among elderly individuals globally and in Saudi Arabia. Factors like health services, lifestyle, and chronic diseases affecting seniors are examined. However, there is a gap in culturally relevant research, particularly in Arabic-speaking countries. This study aims to understand elderly individuals’ knowledge, attitudes, and practices regarding healthy lifestyles for effective functional preservation in aging.

Methodology: A cross-sectional study was conducted in the eastern part of Saudi Arabia, specifically Al-Ahsa, from February to May 2023. The Raosoft calculator was employed to determine a sample size of at least 384 participants. The data was analyzed using SPSS.

Results: Regarding the associations between knowledge levels and demographics, education significantly impacts knowledge (p=0.003). Retired respondents exhibit higher knowledge (50.4%) compared to those with jobs (10.4%) (p=0.002). Smoking has a significant impact on knowledge (p=0.012). Regarding the opinions on elderly care, respondents agree on the importance of fresh fruits and vegetables (52.2%), increased protein intake (64.3%), less fat (83.5%), and regular exercise (44.3%). Supplements’ necessity is disagreed upon (95.7%). Living with family is favored (67.8%), and elderly self-management is recognized (60.9%). Significant differences are seen in fruit and vegetable consumption (p=0.001), less fat usage (p=0.000), exercise habits (p=0.000), smoking (p=0.000), and using just salt in cooking (p=0.000).

Conclusion: Study findings underscore the importance of education in influencing healthy behaviors and informed choices, with education levels significantly impacting knowledge levels. Respondents’ preferences for balanced diets, exercise, and self-management reflect a positive trend toward embracing healthy aging principles. Notably, the study identifies disparities between knowledge groups in various lifestyle factors, highlighting the potential of education to drive positive changes in behaviors.

## Introduction

Most countries define an elderly person as one who is above 60 years old [1]. Healthy lifestyles in the elderly are defined as an ongoing process that involves participation in many aspects of life, including work, fitness, family, and community [2]. The following factors have contributed to an increase in life expectancy for people all over the world: (a) the rapid development of health services, which has improved life quality; (b) healthy eating habits; (c) increased levels of physical activity; and (d) the substantial reduction of smoking and alcohol use [1]. The proportion of senior people in society is rising, which is increasing the demand for healthcare services and other social services [3]. Compared to other different age groups, elderly individuals have the highest prevalence of chronic diseases and the largest, most significant expenses associated with long-term care [4]. Regular physical activities and a multifaceted healthy lifestyle are essential because they can help prevent diseases, manage existing conditions, and improve overall quality of life [5-7]. A person's lifestyle is shaped by their identities and choices; it cannot be studied in separation from their social and cultural environment. That means an individual's daily routine and way of life are influenced by their social community; family; friends; and biological, psychological, and personal characteristics [8].

Studies have examined differences and similarities in healthy lifestyle awareness among older adult populations around the world. There is a growing global interest in collecting gerontological data [9]. The relationship between individual lifestyle factors, health, and functioning in elderly people, including physical activity, smoking habits, and body weight, is well supported by epidemiological studies [4]. These studies, however, only examined one particular lifestyle component, making it difficult to compare different lifestyle factors in terms of how they relate to functioning [4]. Particularly few researchers have focused on how lifestyle factors and a generally healthy lifestyle affect social functioning [4]. In order to formulate concepts for healthy aging, the majority of research on healthy aging has been done in Western and wealthy countries; however, there has not been much culturally pertinent study on healthy aging in Arabic-speaking countries, particularly in Saudi Arabia [10]. Early gerontological studies looked at elderly Saudi Arabian perspectives toward aging, such as the work of Mansour and Laing (1994) [9]. The subjective understanding of Muslim cultures and Arab countries' attitudes on aging and healthy aging, as well as the elements that influence the aging process and healthy aging concepts, must be taken into consideration in order to identify and address the research gaps [10]. Based on this information, specific lifestyle recommendations could be made to certain older persons who are at risk of functional decline. The goal of extending healthy years of life will be helped enormously by this research. We anticipate that there is a lack of awareness and a negative attitude toward healthy lifestyles for older people, and it is a contributing factor in increasing mobility and disability among the elderly. The purpose of this study is to identify the knowledge, attitude, and practice of the elderly toward a healthy lifestyle during aging.

## Materials and methods

A cross-sectional study was conducted in the Eastern part of Saudi Arabia, Al-Ahsa, from February to May 2023 using the Raosoft calculator (Raosoft, Inc., Seattle, WA) for calculating a sample size of at least 384 with a margin error of 5% and a confidence level of 95% to analyze the awareness on healthy lifestyle ‎during aging among Al-Ahsa elderly people. An online validated questionnaire (consisting of questions related to knowledge and attitudes) with Arabic translation was employed and disseminated randomly on social media for two months. The average time expected to fill out the questionnaire is 3-6 minutes‎‏.‏ People from Al-Ahsa who were 60 years old or older were eligible to participate. People from cities other than Al-Ahsa and those under the age of 60 were excluded. 

Sociodemographic data were considered as predictors, while awareness level of unhealthy lifestyles during aging and lack of awareness and knowledge about healthy lifestyles were treated as outcomes. The questionnaire was divided into four sections intended for assessing the elderly awareness of a healthy lifestyle during aging. There are 35 questions that consist of some biographical data, healthy lifestyle knowledge, healthy lifestyle attitude, and some lifestyle practices. The questionnaire results are collected electronically using Google Forms (Google LLC, Mountain View, CA) and Statistical Package for the Social Sciences (SPSS) and by direct interview. The data were analyzed using IBM SPSS, Windows 26.0 edition (IBM, Armonk, NY), and the data was presented on tables and graphs using Microsoft Excel 2016 (Microsoft Corporation, Redmond, WA). The total replies for each choice in the survey were estimated using the frequency of the questions. The major statistical analytical test was chi-square, while the remaining quantitative data was analyzed using one-way ANOVA.

This study did not require financial funding or the use of resources. All participants’ consent was obtained before they were involved in the study. All data were kept confidential, and no subject was identified by name. The study was conducted under the approval of the Ethics Committee of King Faisal University, and the mentor did assure quality, monitoring, and safety.

## Results

The majority of respondents, accounting for 83.8%, fall within the 60-69 age range, followed by age groups 70-79 and above 80 representing smaller proportions at 8.5% and 7.7%, respectively, as shown in Table 1. The table shows that the sample is fairly, evenly split between males and females, with 44.9% and 55.1%, respectively. Moving on to marital status, the data reveals that the largest proportion of respondents, comprising 71.0%, are married. The widowed category represents a significant portion at 18.8%, while single individuals account for 5.9% and divorced individuals makeup 4.4% of the sample. Examining the educational background of the respondents, the data highlights a diverse range. Approximately 17.6% of the sample are illiterate, while 26.1% have education below a diploma. A total of 11.0% possess a diploma, and the majority, 45.2%, have received higher education. The table also includes information about the respondents' living arrangements and the cost of living. It shows that the largest group, comprising 51.8%, is retired. Additionally, 17.6% of the respondents have a job, while 15.8% live with children, and smaller proportions live with relatives (6.6%) or in an institution (8.1%). Finally, the data presents insights into smoking habits. The majority of respondents, accounting for 94.5%, do not smoke, while a smaller proportion, at 5.5%, reported to smoke rarely.

**Table 1:** Demographic data of participants

		n	%
Age	60-69	228	83.80%
	70-79	23	8.50%
	More than 80	21	7.70%
Gender	Male	122	44.90%
	Female	150	55.10%
Marital status	Single	16	5.90%
	Married	193	71.00%
	Widow	51	18.80%
	Divorce	12	4.40%
Education	Illiterate	48	17.60%
	Under diploma	71	26.10%
	Diploma	30	11.00%
	Higher	123	45.20%
Cost of living	With job	48	17.60%
	Retired	141	51.80%
	With children	43	15.80%
	With relatives	18	6.60%
	Institution	22	8.10%
Smoking	Nonsmoker	257	94.50%
	Rarely smoke	15	5.50%

Table 2 shows that there is no significant association between age, gender, marital status, and knowledge levels (p=0.605, 0.113, 0.07, respectively). Regarding the variable of education, the table classifies individuals into four educational levels: illiterate, under diploma, diploma, and higher education. Among the illiterate, 30 out of 115 (26.1%) display good knowledge, whereas among those with under diploma education, 34 out of 121 (29.6%) exhibit good knowledge. Among diploma holders, 10 out of 115 (8.7%) possess good knowledge, and in individuals with higher education, 41 out of 115 (35.7%) demonstrate good knowledge. The p-value associated with education is 0.003, indicating a significant association between education level and knowledge. Shifting to the variable cost of living, individuals are categorized based on their employment and living situation. Among those with a job, 12 out of 115 (10.4%) have good knowledge, whereas among retired individuals, 58 out of 115 (50.4%) possess good knowledge. The proportion of good knowledge among individuals living with children is 20 out of 115 (17.4%), and for those living with relatives, it is eight out of 115 (7%). Among individuals living in institutions, 17 out of 115 (14.8%) exhibit good knowledge. The p-value for cost of living is 0.002, indicating a significant association between cost of living and knowledge levels. Finally, the variable smoking categorizes individuals into two groups: nonsmokers and those who rarely smoke. Among nonsmokers, 104 out of 115 (90.4%) demonstrate good knowledge, while among rarely smoking individuals, 11 out of 115 (9.6%) possess good knowledge. The p-value associated with smoking is 0.012, indicating a significant association between smoking habits and knowledge levels.

**Table 2:** Demographic data associated with knowledge of participants **Significant at 0.0 *Significant at 0.05

		Knowledge				p-value
		Good		Poor		
Age	60-69	95	82.6%	133	84.7%	0.605
	70-79	9	7.8%	14	8.9%	
	More than 80	11	9.6%	10	6.4%	
Gender	Male	58	50.4%	64	40.8%	0.113
	Female	57	49.6%	93	59.2%	
Marital status	Single	4	3.5%	12	7.6%	0.07
	Married	78	67.8%	115	73.2%	
	Widow	29	25.2%	22	14.0%	
	Divorce	4	3.5%	8	5.1%	
Education	Illiterate	30	26.1%	18	11.5%	0.003**
	Under diploma	34	29.6%	37	23.6%	
	Diploma	10	8.7%	20	12.7%	
	Higher	41	35.7%	82	52.2%	
Cost of living	With job	12	10.4%	36	22.9%	0.002**
	Retired	58	50.4%	83	52.9%	
	With children	20	17.4%	23	14.6%	
	With relatives	8	7.0%	10	6.4%	
	Institution	17	14.8%	5	3.2%	
Smoking	Nonsmoker	104	90.4%	153	97.5%	0.012*
	Rarely smoke	11	9.6%	4	2.5%	

Table 3 presents data on various opinions and attitudes toward specific aspects of elderly care and lifestyle. The first topic addressed is the importance of daily consumption of fresh fruits and vegetables in the diet program for the elderly. The majority of respondents (52.2%) who expressed an opinion on this matter agreed that it is necessary, while a smaller proportion disagreed (18.3%) or had no idea (29.6%). Notably, a statistically significant p-value of 0.007 indicates a relationship between knowledge and the importance placed on fresh fruits and vegetables.

**Table 3:** Opinions and attitudes toward specific aspects of elderly care and knowledge **Significant at 0.01 *Significant at 0.05

		Knowledge				p-value
		Good		Poor		
		n	%	n	%	
Daily usage of fresh fruits and vegetables is the elderly’s diet program in necessary	Agree	60	52.2%	109	69.4%	0.007**
	Disagree	21	18.3%	24	15.3%	
	No idea	34	29.6%	24	15.3%	
Using more protein (low-fat meat, white meat, low-fat or skim milk, and cereals) is necessary for the elderly	Agree	74	64.3%	134	85.4%	0.0001**
	Disagree	6	5.2%	15	9.6%	
	No idea	35	30.4%	8	5.1%	
Diet in elderly should have less fat than younger people	Agree	96	83.5%	148	94.3%	0.014*
	Disagree	12	10.4%	5	3.2%	
	No idea	7	6.1%	4	2.5%	
Regular physical exercise in old age is necessary	Agree	51	44.3%	115	73.2%	0.0001**
	Disagree	15	13.0%	24	15.3%	
	No idea	49	42.6%	18	11.5%	
If proper diet is used, there is no necessity for supplements	Agree	110	95.7%	154	98.1%	0.46
	Disagree	4	3.5%	2	1.3%	
	No idea	1	0.9%	1	0.6%	
Living with the family is necessary for the elderly	Agree	78	67.8%	106	67.5%	0.572
	Disagree	27	23.5%	42	26.8%	
	No idea	10	8.7%	9	5.7%	
Elderly should manage their life in old age	Agree	70	60.9%	120	76.4%	0.006**
	Disagree	45	39.1%	37	23.6%	

The next topic discussed is the inclusion of more protein, such as low-fat meat, white meat, low-fat or skim milk, and cereals, in the elderly diet. A majority of respondents (64.3%) agreed that this is necessary, while only a small percentage disagreed (5.2%) or had no idea (30.4%). The corresponding p-value of 0.0001 suggests a strong association between knowledge and the perceived necessity of increased protein intake.

The table also explores the belief that the diet of elderly individuals should have less fat compared to younger people. A significant majority (83.5%) agreed with this statement, while a smaller proportion disagreed (10.4%) or had no idea (6.1%). The associated p-value of 0.014 indicates that knowledge may influence the perception of fat intake in older adults.

The importance of regular physical exercise in old age is another topic addressed. While a substantial number of respondents (44.3%) agreed that it is necessary, a significant proportion (42.6%) had no idea about this matter. However, the majority of those who expressed an opinion agreed with the statement, and the p-value of 0.0001 signifies a strong relationship between knowledge and the recognition of regular exercise as important for the elderly.

Regarding the necessity of supplements when a proper diet is followed, an overwhelming majority (95.7%) agreed that supplements are not necessary. A small percentage disagreed (3.5%) and an even smaller proportion had no idea (0.9%). The non-significant p-value of 0.46 suggests that knowledge does not strongly correlate with the belief in the necessity of supplements.

The topic of living arrangements for the elderly is also discussed. The majority of respondents (67.8%) agreed that living with family is necessary, while a slightly smaller percentage disagreed (23.5%) or had no idea (8.7%). The p-value of 0.572 indicates that knowledge does not significantly impact the opinion on this matter.

Finally, the belief that the elderly should manage their lives in old age is examined. A majority (60.9%) agreed with this statement, while a notable proportion (39.1%) disagreed. The corresponding p-value of 0.006 suggests a significant relationship between knowledge and the perception of elderly individuals managing their lives.

Table 4 provides insights into various lifestyle factors and their association with knowledge levels. Regarding the consumption of liquids, there is no significant difference between individuals with good knowledge and poor knowledge (p=0.447). Both groups reported similar percentages of individuals who drink the recommended amount of six to eight glasses per day. However, when it comes to the consumption of fruits and vegetables, a significant difference emerges (p=0.001). Individuals with poor knowledge tend to consume enough fruits and vegetables at a higher rate compared to those with good knowledge.

**Table 4:** Lifestyle factors of participants and their association with knowledge levels **Significant at 0.01

		Knowledge				p-value
		Good		Poor		
		n	%	n	%	
Do you drink 6-8 glasses of liquid in a day?	Yes	84	73.0%	108	68.8%	0.447
	No	31	27.0%	49	31.2%	
Do you use enough fruits and vegetables daily?	Yes	42	36.5%	90	57.3%	0.001**
	No	73	63.5%	67	42.7%	
Do you use more protein (low-fat meat, white meat, low-fat or skim milk, and cereals) than other members of the family?	Yes	113	98.3%	147	93.6%	0.066
	No	2	1.7%	10	6.4%	
Do you live with a family member? (yes or no)	Yes	46	40.0%	102	65.0%	0.000**
	No	69	60.0%	55	35.0%	
Do you use less fat than other members of a family?	Yes	20	17.4%	70	44.6%	0.000**
	No	95	82.6%	87	55.4%	
Do you use supplements along with meals?	Yes	83	72.2%	125	79.6%	0.153
	No	32	27.8%	32	20.4%	
Do you use just the salt in food while cooking?	Yes	24	20.9%	76	48.4%	0.000**
	No	91	79.1%	81	51.6%	
Do you exercise regularly and daily?	Walking	53	46.1%	110	70.1%	0.000**
	Running	0	0.0%	3	1.9%	
	Other	11	9.6%	8	5.1%	
	Do not do exercise	51	44.3%	36	22.9%	
How do you exercise daily?	10 min	5	4.3%	20	12.7%	0.000**
	15 min	12	10.4%	15	9.6%	
	20 min	8	7.0%	18	11.5%	
	Do not do exercise	63	54.8%	40	25.5%	
	30 min	17	14.8%	42	26.8%	
	More than 5 min	10	8.7%	22	14.0%	
Do you have enough sleep?	Yes	97	84.3%	123	78.3%	0.214
	No	18	15.7%	34	21.7%	
Do you smoke?	Yes	37	32.2%	93	59.2%	0.000**
	No	78	67.8%	64	40.8%	
Do you have active participation in social activities?	Yes	63	54.8%	99	63.1%	0.170
	No	52	45.2%	58	36.9%	
Do you visit your doctor at least every 6 months?	Yes	63	54.8%	99	63.1%	0.170
	No	52	45.2%	58	36.9%	

Protein intake does not exhibit a significant disparity between the two knowledge groups (p=0.066). The majority of respondents in both groups reported consuming more protein than other family members. Living arrangements have a notable impact on knowledge levels (p=0.000). Individuals living with family members have a lower percentage of good knowledge compared to those not living with family members. The use of less fat in food preparation showcases a significant difference between the knowledge groups (p=0.000). Individuals with poor knowledge are more likely to use less fat compared to other family members. Supplement usage does not vary significantly based on knowledge levels (p=0.153). Both groups reported relatively similar percentages of individuals using supplements with their meals.

The use of just salt in cooking demonstrates a significant difference between the knowledge groups (p=0.000). Individuals with poor knowledge tend to rely more on salt as the sole seasoning in their food preparation. Exercise habits vary significantly between individuals with good knowledge and poor knowledge (p=0.000). Those with poor knowledge engage in regular exercise, especially walking, at a higher rate compared to individuals with good knowledge. The duration of daily exercise also differs between the two knowledge groups (p=0.000). Individuals with good knowledge exhibit a more balanced distribution across different exercise durations, while individuals with poor knowledge are more likely to report not engaging in any exercise. Sufficient sleep does not significantly differ between the knowledge groups (p=0.214). Both groups report a high percentage of individuals claiming to have enough sleep.

Smoking habits reveal a significant difference between individuals with good knowledge and poor knowledge (p=0.000). A higher percentage of individuals with poor knowledge reported smoking compared to those with good knowledge. Active participation in social activities does not exhibit a significant disparity between the knowledge groups (p=0.170). Both groups reported relatively similar percentages of individuals actively participating in social activities. Finally, visiting a doctor at least every six months does not significantly differ between individuals with good knowledge and poor knowledge (p=0.170). Both groups reported similar percentages of individuals adhering to regular doctor visits.

## Discussion

In addition to improving well-being and engagement as people age, maintaining a healthy lifestyle can lower the chance of developing chronic diseases and lower the recovery time. A healthy lifestyle is typically thought to be a manner of life that lowers the risk of developing diseases and prevents early deaths [8].

According to our research results, most of the participants were between the ages of 60 and 69, while those who were 70 or older responded less. According to Saudi Arabia Social Media Statistics 2023, the utilization of social networking sites is lower among persons over the age of 70, which may help to explain this considering that the questionnaire was distributed through social media platforms [11].

The study demonstrates no correlation between age, gender, marital status, and knowledge, while there was a significant correlation between education, cost of living, smoking, and knowledge. Findings from the present study revealed that elderly overall knowledge was significantly lower among those who are aged above 70 years, smokers, and unemployed and higher among those who are not smokers and have a high level of education. The result was predictable. Age and education levels are the most important demographics influencing a person's ability to assess their health. Based on epidemiological research, higher levels of education, income, and nonsmoker status are associated with higher self-ratings of health [12]. These outcomes correspond with findings from various national and international studies. In Iran, Taheri reported that there is a relationship between educational level, aging, and knowledge about a healthy lifestyle; the average knowledge score of the studied population has decreased with increasing age [7]. In view of chronic diseases and physical conditions more often observed in older people, they may perceive their health to be worse than younger people. A study published by Kunst indicated that health perception decreased with a decreased level of income [13]. The income level is not used as frequently as an indicator of education or employment status; however, it can provide information on access to goods and services such as the quality of training and health care [12]. When compared to nonsmokers, current smokers have a worse self-perception of their health. This could be due to older age, a lower level of education, a higher likelihood of chronic diseases, or a combination of these factors. The Polish National Health Program 2007-2015 identified the listed determinants of health, such as smoking status and physical activity, as important priority goals, indicating that all efforts should be made to end cigarette smoking and environmental tobacco smoke exposure and to increase recreational physical activity [12]. Elderly people are also targeted by the national health program for ensuring a healthy environment and opportunities to live healthy lives. This analysis supports the theory that there is a lack of awareness towards healthy lifestyles for older people.

This study has underscored the different associations of sociodemographic background, attitudes, practices, and knowledge with well-being in old age. In Saudi Arabia, there are few studies on aging [10]. Therefore, it is necessary to undertake further research in particular studies focusing on the perspective of healthy aging from a Saudi Arabian elderly population. For the prevention of diseases and disabilities, it is generally more efficient to adopt a healthier lifestyle as an early adult and continue into older ages. There is a significant relationship between elderly individuals who daily use fresh fruit and vegetables and those who do not, which indicates that knowledge levels play a vital part in consuming fruit and vegetables in an elderly population. This spotting aligns with another research, which noted relative connections between high perception and dietary habits including fresh fruit and vegetables [14]. Moreover, we find that there is a strong significant role between using more protein and the elderly's knowledge, which is also supported by a similar study report that there is a significant relationship between high knowledge and using more protein diet [15]. Our study found that there was an impact of less fat consumption and higher knowledge levels on healthy lifestyle awareness, which suggests that there is an association between the elderly’s knowledge and having less fat compared to younger people. This result has been given in another research that reported that those who have high knowledge have a less fat diet [7].

When it comes to regular physical exercise, we found a potent association between the elderly's knowledge and physical exercise. This finding adjusts to an existing study, which reported that there is a significant relationship between regular physical exercise and knowledge levels [3].

In our study, we found an interesting spotting that the majority of elderly believe they should manage their lives in old age. They still believe their lives should be handled on their own despite the obstacles, diseases, and fatigue they might have [7].

Regarding the consumption of liquids, our study found no significant difference between individuals with good knowledge and poor knowledge. This suggests that knowledge levels may not play a crucial role in influencing the intake of liquids among the study population. This finding is in line with a study, which also reported no significant association between knowledge levels and liquid consumption in a similar demographic [16].

However, when considering the consumption of fruits and vegetables, a significant difference emerged between the two knowledge groups. Individuals with poor knowledge tended to consume enough fruits and vegetables at a higher rate compared to those with good knowledge. This result is intriguing and contradicts the common perception that higher knowledge levels are directly linked to better dietary habits [17]. Regarding protein intake, it was observed that there was no significant disparity between the two knowledge groups. This suggests that knowledge levels may not be a critical factor influencing protein consumption habits among the study population. However, it is essential to note that this finding is not consistent with the prior research, which reported a positive correlation between knowledge levels and protein intake among a comparable sample [18].

Living arrangements were found to have a notable impact on knowledge levels. Individuals living with family members had a lower percentage of good knowledge compared to those not living with family members. This finding is particularly interesting, as one might expect that living with family could facilitate information sharing and possibly lead to higher knowledge levels [19]. The use of less fat in food preparation showed a significant difference between the knowledge groups. Individuals with poor knowledge were more likely to use less fat compared to other family members. This finding aligns with existing studies, which reported similar associations between knowledge levels and fat consumption patterns [20].

Supplement usage, on the other hand, did not vary significantly based on knowledge levels. Both groups reported relatively similar percentages of individuals using supplements accompanying their meals. This result indicates that knowledge levels may not play a substantial role in shaping supplement consumption behavior among the study population [21]. The use of just salt in cooking exhibited a significant difference between the knowledge groups, with individuals with poor knowledge relying more on salt as the sole seasoning in their food preparation. This result raises concerns about potential health implications, as excessive salt consumption is linked to various health issues [22].

Exercise habits emerged as a crucial lifestyle factor associated with knowledge levels. Individuals with poor knowledge engaged in regular exercise, especially walking, at a higher rate compared to individuals with good knowledge. Additionally, the duration of daily exercise differed significantly between the two knowledge groups, with individuals with good knowledge exhibiting a more balanced distribution across different exercise durations. This finding highlights the need for targeted health education programs to address the potential misconception that higher knowledge levels alone are sufficient for promoting healthy behaviors.

Sufficient sleep did not significantly differ between the knowledge groups, with both reporting a high percentage of individuals claiming to have enough sleep. This result suggests that knowledge levels may not be a determining factor in achieving adequate sleep duration. Smoking habits showed a significant difference between individuals with good knowledge and poor knowledge, with a higher percentage of individuals with poor knowledge reporting smoking. This finding is concerning and highlights the urgent need for targeted public health campaigns and interventions to address smoking behaviors, particularly among individuals with lower knowledge levels [23].

Active participation in social activities did not exhibit a significant disparity between the knowledge groups. Both groups reported relatively similar percentages of individuals actively participating in social activities. This result suggests that knowledge levels may not play a critical role in shaping social engagement behaviors among the study population. Finally, visiting a doctor at least every six months did not significantly differ between individuals with good knowledge and poor knowledge. Both groups reported similar percentages of individuals adhering to regular doctor visits. This result implies that knowledge levels may not be a primary driver of health-seeking behaviors, as both groups demonstrated similar rates of doctor visits.

Limitations

Several limitations should be acknowledged in this study. First, the cross-sectional design prevents establishing causal relationships, limiting the ability to ascertain the direction of influence between knowledge and lifestyle factors. Additionally, the reliance on self-reported data might introduce recall and social desirability biases, potentially affecting the accuracy of responses. The study’s location within one specific region of Saudi Arabia might limit the generalizability of the findings to the broader population. Furthermore, the study’s dependence on a specific age group (60 years and above) excludes insights from younger individuals who might exhibit different behaviors and attitudes.

## Conclusions

Study findings underscore the importance of education in influencing healthy behaviors and informed choices, with education levels significantly impacting knowledge levels. Respondents’ preferences for balanced diets, exercise, and self-management reflect a positive trend toward embracing healthy aging principles. Notably, the study identifies disparities between knowledge groups in various lifestyle factors, highlighting the potential of education to drive positive changes in behaviors.
